# The Value of Management in the Workroom

**Published:** 1912-02-03

**Authors:** 


### INSTITUTIONAL HOUSEKEEPING.

(Workers' Questions and Criticism of every kind will be welcomed.)

### The Value of Management in the Workroom.

The management of the workroom of an institution is an important part of the work of the matron, housekeeper, or sister-in-charge. The work of this department starts with establishing good methods in sorting and mending the linen as it is received from the laundry at definite intervals, and in the systematic renewal of all worn-out articles.

In large hospitals there is usually a special sister who superintends the workroom; in smaller institutions it is under the care of the matron, who arranges that her own maid or the housekeeper shall help in the mending and see that it is properly carried out.

All linen from the counterpanes to the dusters should be distinctly marked with the name or number of the ward, and the date when new. Often a special number is given so that sheets, etc., may be used in rotation, and if any article is lost the exact one missing may be detected. The marking should be done in the workroom, unless undertaken by the shop, which will usually mark all the large things, sheets, and counterpanes, etc. The marking should be large and clear, and to this end good marking ink must be obtained, and the directions supplied with it carefully observed. Many of the modern kinds wash out or make holes. Marking in cotton is such a tedious process that though most durable it cannot be recommended.

The linen leaves the laundry after being sorted into separate baskets, which are conveyed to the linen room of the institution or to the linen cupboard attached to each ward.

The ward sister or a responsible probationer looks over the linen with great care. In the women's wards the mending is sometimes done by the women patients, but even in this case all the torn articles are sent to the workroom to be looked over and redistributed. This may be done every week, but if the linen be carefully mended, once a fortnight may suffice. In some of the smaller institutions the night nurses help in the mending. In others there is a sewing committee and a work party connected with it, the members of which undertake the renewal of all the house linen, the thrifty use of old blankets, sheets, etc., and sometimes even the mending.

In the workroom there is a sewing maid, whose duty it is to mend, or prepare for others to mend, all the linen sent to her, and to sort and look over all that does not come under the care of the sisters of the wards and operating theatre. Other maids in the hospital—for example, the dining-room maids, the matron, and house-surgeon's maids—should help in the workroom in the afternoon, but this should always be clearly understood on their engagement, and the sewing hours noted down in their timetable. There must always be a responsible person in the workroom capable of superintending the work of the younger and less experienced, and of seeing that the time arranged for is profitably spent. The sewing-room maid ought to be capable of this superintendence, but otherwise it would devolve on the matron's maid, who would naturally have a superior position to the other domestics.

The sewing-room maid must have thorough knowledge of mending, and must be put on her mettle to make a thrifty use of old sheets, blankets, etc., too worn-out for their original use. Thin worn-out sheets placed together, cut to convenient sizes and machined round, make useful dusters and polishing cloths. Worn sheets can be split, and the less worn-out sides placed to the middle and machined as flat as possible. Two worn blankets placed together and quilted across can take the place of one good one. Tapes should always be sewn on new towels, dusters, etc., to prevent tearing at the corners. If the workroom is carefully managed more care will be taken of the various articles in use. Dusters will not be allowed to get ragged, or used for wrong purposes; tea-cloths and glass-cloths will be respected; for if any ward is especially careless over the linen belonging to it, the misuse will be brought to the notice of the ward sister.

The management of this part of the work of the hospital differs much in various institutions, but perhaps the chief fault in all is the lack of strict supervision. The linen must pass through many hands, and there is always so much to do, and so little time to do it in, that it is most important someone in authority should keep an eye on every article in use, and that mending and checking should be carried out with great exactitude at regular intervals.

In many institutions the uniforms of the hospital staff and the servants are made in the workroom. The number of hands would then be increased, and a practical dressmaker would be placed at the head of the department. If possible a dressmaker should be engaged who has had institution experience, and large, well-managed workrooms, such as are run at Guy's Hospital, train good applicants for these posts. The workroom should contain no furniture beyond chairs, tables, large and small, cupboards, and presses. A good sewing-machine, periodically overhauled, is a first necessity. A gas ring for heating irons, and an ironing blanket for pressing some of the mended articles that require it are also useful.

At certain times of the year, usually about July, when hospital work is less, and spring cleaning is over, there should be an annual inspection of all the house linen by the matron or housekeeper, and every article in use should be looked over, and, if worn out, discarded or used up in other ways. A requisition list should be made of all articles required, including reels of cotton, needles, tapes, and other mending materials. All stores should be replenished, and numbers made up. The choice of materials is a matter of great importance, demanding judgment and experience, nor should the selection ever be made in a hurry.

